# Natural genetic variation in Stim1 creates stroke in the spontaneously hypertensive rat

**DOI:** 10.1038/s41435-020-0097-5

**Published:** 2020-04-17

**Authors:** Isha S. Dhande, Sterling C. Kneedler, Yaming Zhu, Aniket S. Joshi, M. John Hicks, Scott E. Wenderfer, Michael C. Braun, Peter A. Doris

**Affiliations:** 1 Institute of Molecular Medicine, University of Texas Health Science Center at Houston, Houston, TX 77030 USA; 2 Department of Pathology and Immunology, Baylor College of Medicine and Texas Children’s Hospital, Houston, TX 77030 USA; 3 Department of Pediatrics, Baylor College of Medicine and Texas Children’s Hospital, Houston, TX 77030 USA

**Keywords:** Lymphocytes, Disease genetics

## Abstract

Similar to humans, the risk of cerebrovascular disease in stroke-prone spontaneously hypertensive rats (SHR-A3/SHRSP) arises from naturally occurring genetic variation. In the present study, we show the involvement of genetic variation affecting the store-operated calcium signaling gene, *Stim1*, in the pathogenesis of stroke in SHR. *Stim1* is a key lymphocyte activation signaling molecule and contains functional variation in SHR-A3 that diverges from stroke-resistant SHR-B2. We created a SHR-A3 congenic line in which *Stim1* was substituted with the corresponding genomic segment from SHR-B2. Compared with SHR-A3 rats, *Stim1* congenic SHR-A3 (SHR-A3(*Stim1*-B2)) have reduced cerebrovascular disease in response to salt loading including lower neurological deficit scores and cerebral edema. Microbleeds and major hemorrhages occurred in over half of SHR-A3 rats. These lesions were absent in SHR-A3(*Stim1*-B2) rats. Loss of *Stim1* function in mice and humans is associated with antibody-mediated autoimmunity due to defects in T lymphocyte helper function to B cells. We investigated autoantibody formation using a high-density protein array to detect the presence of IgG and IgM autoantibodies in SHR-A3. Autoantibodies to key cerebrovascular stress proteins were detected that were reduced in the congenic line.

## Introduction

Hypertension is a primary risk factor for cerebrovascular disease and stroke [1], both of which are associated with significant morbidity and mortality. Susceptibility to stroke has a substantial heritable component [2–4] though the underlying genetic risk pathways leading to stroke in the presence of hypertension are not known. Genetic complexity and gene-environment interactions pose difficulties for genetic epidemiological studies in human subjects. The stroke-prone spontaneously hypertensive rat (SHR-A3, SHR-SP) is a well-characterized inbred genetic model of cerebrovascular disease [5–7]. Similar to humans, risk of cerebrovascular injury, including cerebral edema, microbleeds, and major hemorrhages, is affected in SHR-A3 rats by naturally occurring genetic variations [6, 8]. SHR-A3 and the closely related SHR-B2 line were produced by selective breeding on the trait of elevated blood pressure starting in the 1950’s [9]. Both SHR lines are descended from the same founder pair of outbred Wistar rats and subsequent inbred progeny of these founders provided the progenitors of both SHR-A3 and SHR-B2. SHR-B2 and other SHR lines resist cerebrovascular disease and stroke. The first observation of cerebrovascular disease in the SHR-A3 line occurred after 20 generations of inbreeding, suggesting the possible emergence of a new mutation [10]. Further selective breeding was used to fix genetic variation responsible for the stroke-susceptibility trait. Cerebrovascular disease in SHR-A3 reduces life span in this line from the ~100 weeks life span of SHR-B2 to ~40 weeks, or less with increased salt intake.

Genetic variation in the genomes of SHR-A3 and SHR-B2 reflects their recent common ancestry (shared founder animals and eight generations of inbreeding before separation of the lines) and results in 87% genetic identity by descent [11]. Gene variation affecting end-organ disease may arise in ancestral variation in the 13% of the genome by which these lines differ, or as a result of de novo mutation arising after separation of the lines. Since stroke susceptibility is polygenic [12], both ancestral and de novo genetic variation may contribute. Our prior analysis of whole-genome sequences of SHR-A3 and SHR-B2 revealed a deleterious mutation in a ~45Mbase haploblock on chromosome 1 of SHR-A3, that is, otherwise identical to SHR-B2 [13]. This appears to be a recent mutation unique to the SHR-A3 line. It affects the *Stim1* gene and is present in all 3 SHR-A3 colonies for which genome sequence is available, but absent in other inbred rat lines [14]. It creates a premature stop codon that results in expression of a truncated form of the stromal interaction molecule 1 (STIM1) protein. STIM1 is an ER Ca^2+^ sensor essential for store-operated calcium entry (SOCE) [15]. Loss of SOCE impairs follicular T lymphocyte activation, with numerous functional defects including spontaneous autoantibody production and humoral autoimmunity [16].

In the present study, we have tested the effect of variation in *Stim1* on T-cell function in SHR-A3 by creating a congenic substitution line in which the defective SHR-A3 *Stim1* gene is rescued by transfer from SHR-B2. We have also examined the emergence of cerebrovascular disease in the Stim1-rescued SHR-A3 line and compared it with SHR-A3. Because Stim1 deficiency creates a spontaneous autoimmune phenotype in humans, we have assessed the indicators of autoantibody production and we have performed an autoantigen discovery study using a novel proteomic array technology. This comprises an array printed with ~20,000 unique recombinant human proteins that we used to evaluate the presence and targets of self-reactive antibodies in SHR-A3 and in an SHR-A3 congenic line which contained functional *Stim1*.

## Methods

The authors declare that all supporting data are available within the article [and its online supplementary files].

### Animals and treatments

The Institutional Animal Welfare Committee prospectively reviewed and approved all animal experiments and protocols. Studies were performed on male animals from the injury-susceptible spontaneously hypertensive–A3 (SHR-A3, SHRSP/Bbb) and the injury resistant SHR-B2 rat lines, maintained in our AAALAC-approved specific pathogen-free facility. Female SHR-A3 rats have a low rate of cardiovascular end-organ disease and were not included in this study. These lines and their origins prior to transfer to our laboratory have been recorded at the Rat Genome Database (rgd.mcw.edu) which has applied the following identifiers: SHR-A3 – RGD ID = 8142383, Symbol = SHRSP/BbbUtx; SHR-B2 – RGD ID = 8142385, Symbol = SHR/Utx. Animals were provided a standard rodent chow diet and drinking water ad libitum.

### *Stim1* congenic line creation

SHR-A3 *Stim1* mutation exists within an extended haplotype block of genetic identity in which background genetic variation from a single common ancestor has been fixed in both SHR-A3 and SHR-B2. The *Stim1* encompassing block extends for ~45 Mb on chromosome 1 (chr1:163Mb-208Mb) and, except for the *Stim1* mutation, contains no other non-synonymous coding sequence variants between SHR-A3 and SHR-B2. SHR-A3 (males) and SHR-B2 (females) parental line animals were crossed to generate the F1 progeny. This progeny was backcrossed into SHR-A3 animals for five generations. Backcrossed animals were genotyped at each generation using a previously described panel of ~200 single nucleotide polymorphism (SNP) markers to allow speed congenic selection of optimal animals (highest loss of SHR-B2 background alleles while retaining the introgressed SHR-B2 *Stim1* haplotype block). The final congenic line was created by mating heterozygous male and female animals from the previous backcross and selecting their progeny that were homozygous for SHR-B2 alleles at the *Stim1* locus and for SHR-A3 alleles at any locus at which heterozygosity remained in the prior backcross. Since the *Stim1* mutation lies in a region that is identical by descent between SHR-A3 and SHR-B2, the resulting congenic is genetically identical to SHR-A3 line with the exception of the functional *Stim1* gene.

### Peripheral blood mononuclear cell and lymphocyte isolation

Peripheral blood was collected from the abdominal aorta of isoflurane-anesthetized rats. Lymphocytes were isolated from whole blood using Lymphocyte Separation Medium (LSM) (Lonza, Allendale, NJ) according to the manufacturer’s directions. Briefly, whole blood was diluted 1:1 with sterile buffered saline and layered on top of LSM at a ratio of 3:2 followed by centrifugation at 400 × *g* for 20 min at 4 °C. Lymphocytes, were collected and washed twice followed by centrifugation at 70 × *g* for 10 min to remove platelets. For isolation of splenocytes, spleens were cut into small fragments and passed through a 70 μm cell strainer into a 50 mL conical tube. Collected cells were washed and the pellet was re-suspended in 5 mL erythrocyte lysis buffer and lysis was carried out for 10 min at 25 °C with gentle shaking. After red cell lysis, splenocytes were collected by centrifugation. Lymphocytes were collected from abdominal aortic lymph nodes in a similar manner. Cells were either re-suspended in complete RPMI-1640 (cRMPI containing 10% FBS, 100 U/ml Penicillin-Streptomycin, 4mM L-Glutamine, 1 mM sodium pyruvate, 1% non-essential amino acids, 1% RPMI vitamins, 10 mM HEPES, and 50 μmol/L β-mercaptoethanol), or in flow cytometry staining (FACS) buffer (Biolegend), based on downstream applications.

### Store-operated Ca^2+^ entry

Flow cytometry with the non-ratiometric dye Fluo-3AM (Promokine) was used to assess SOCE in peripheral blood mononuclear cells. Cells were loaded with Fluo-3AM (final concentration 1 μmol/L) for 30 min at 37 °C in cRPMI. Cells were washed and re-suspended in 2 mL Ca^2+^-free Ringers solution. Fluorescence measurements were acquired on a FACSCalibur (BD Biosciences) flow cytometer. Baseline Ca^2+^ measurements were made for 2 min followed by the addition of the ER Ca-ATPase inhibitor thapsigargin (2 μmol/L) for 5 min. SOCE was induced by the addition of 2 mM Ca^2+^ and data were recorded for 5 min. For TCR-induced SOCE, labeled lymphocytes were incubated with biotin-anti-CD3 (1:1000, BD Biosciences) for 5 min prior to baseline measurements. After recording the baseline for 2 min, CD3 was cross-linked by streptavidin (1:3000, ThermoFisher Scientific) followed by the addition of 2 mM Ca^2+^ for 5 min to induce SOCE. Data were analyzed using FlowJo (Treestar) software. EGTA and ionomycin (2 μmol/L) were used to calculate maximal and minimal fluorescence values of Ca^2+^ and actual [Ca^2+^]_i_ was calculated according to the equation: [Ca^2+^]_i_ = Kd [(F (Fmin)/(Fmax (F))].

### NFAT Nuclear Translocation

CD3^+^ T cells (1 × 10^6^ cells/ml) were stimulated with phorbol myristate acetate (PMA, 10 nmol/L) and Ionomycin (2 μmol/L) for 1 h at 37 °C. Following treatment, cells were fixed with 4% paraformaldehyde in 0.1 Phosphate Buffer (Electron Microscopy Sciences) for 10 min. Lymphocytes were then centrifuged in a cytospin for 5 min at 800 rpm onto slides. Cells were then permeabilized by a 5 min incubation with 0.5% Triton X-100 in PBS, followed by washing in PBS for 5 min. Nonspecific binding was blocked with Protein Block (Biogenex) for 1 h at room temperature. Cells were incubated with anti-NFATc1 clone-7A6 (Biolegend) overnight at 4 °C followed by washing and a 1 h incubation at room temperature with Alexa 488-conjugated goat anti-mouse IgG (Biolegend-405319). Nuclear counterstaining was performed with 5 mM Draq5 (Cell Signaling-4084S) for 1 h. Images were acquired with a 63× oil immersion objective (NA1.4) of a Leica TCS SP5 confocal microscope. Single optical sections were obtained with high numerical aperture lens (63× with an additional two times software zoom) to determine the percentage of NFATc1-nuclear cells. At least 200 cells were analyzed from each group with three independent replicates.

### Cytokine production in stimulated T cells

T cells (2 × 10^5^ cells) sorted by FACS from peripheral blood were seeded into 96-well plates and stimulated with PMA (10 nmol/L) and Ionomycin (2 μmol/L) (both from eBiosciences) for 4 h at 37 °C. ELISA was used to quantify IL-2 levels in the cell culture supernatants according to manufacturer’s protocols (R&D Systems anti-rat Quantikine ELISA kit, cat# R2000). Briefly, plates were coated with primary antibodies to IL-2 followed by incubation with cell culture supernatants for 2 h at 25 °C. The total bound antigen was quantified using biotinylated secondary antibodies (1:1000) and visualized using the HRP/Streptavidin-TMB system.

### Blood pressure (BP) measurement

At 16–18 weeks of age, male SHR-A3 and SHR-A3(*Stim1*-B2) rats were implanted with radio-telemetry devices (Data Sciences, St. Paul, MN) to record BP as described previously [17]. Animals were allowed to recover for 2 weeks before initiating BP recordings at age 20 weeks. Baseline BP was recorded 24 h before salt loading. Subsequent BP measurements were taken 1 day per week throughout the study. BP was measured by continuous sampling for 30 s every 30 min for 24 h.

### Stroke induction and assessment

Salt loading (1% NaCl in drinking water, standard 0.4% Na rat chow) was used to accelerate the development of cerebrovascular lesions and was initiated at age 20 weeks, 2 weeks after telemetry probe implantation surgery. Rats were weighed twice per week to monitor stroke-onset. Animals exhibiting rapid weight loss, loss of coordination, reduced motor activity, paralytic gait, and sudden death were considered stroke-sign positive. The Yamori classification scheme was used to score neurological symptoms and stroke severity at euthanasia [5, 18]. Score 1—normal, 2—hyperirritability, aggressiveness, piloerection, hyperkinesia, and jumping, 3—lethargy with hypersensitivity to painful stimuli, and 4—lethargy with hyposensitivity to painful stimuli, akinesia, paralytic gait, and emaciation. Rats losing over 15% of their highest body weight were euthanized. At the end of the treatment, rats were perfused transcardially (under 2.5% isofluorane) with saline followed by 4% buffered formalin. Perfusion pressure was maintained constant at ~80 mmHg. Brains were fixed in 4% buffered formalin for 24 h. Brain weight, and maximal transverse and coronal diameters were measured following fixation. The brains were sectioned into 2 mm slices using a rat brain matrix (Kent Scientific) and gross morphological assessments were performed to detect microbleeds and major hemorrhages following our previously described methods [18].

### HuProt antigen microarray studies

These studies were performed in the Genomics and Microarray Core Facility, University of Texas Southwestern Medical School. HuProt v3.1 arrays (CDI Laboratories, Mayaguez, PR, USA) spotted in duplicate with ~20,000 full-length, well-folded, recombinant human proteins representing >80% of the human proteome were used according to the manufacturer’s protocol. Each individual array was HuProt array was blocked 5% BSA in 1x TBS-T followed by incubation with pooled serum collected from six individual animals SHR-A3 animals aged 16 weeks and consuming normal 0.4% Na content chow and drinking water. Pools were also created from six animals that had drinking water replaced with 1% NaCl at 20 weeks of age using serum collected after 8 weeks of salt loading or serum obtained prior to euthanasia for animals exhibiting significant neurological injury. Similar serum pools were also analyzed from 28-week-old SHR-A3(*Stim1*-B2) congenic animals at the conclusion of 8 weeks of salt loading. After incubation with serum and rinsing, arrays were developed with detection antibodies targeting rat IgM and rat IgG (Goat polyclonal anti-Rat IgM-Heavy chain conjugated with Alexa Fluor 647, Invitrogen A21248, and Goat polyclonal anti-Rat IgG (H + L) conjugated with Cyanine3, Invitrogen A10522). Arrays were further rinsed and fluorescence signals were obtained using a fluorescent slide reader (GenePix^®^ 4400 A Microarray Scanner). Z score normalization was used to permit comparison of autoantibody signals across arrays. Intra-array reproducibility was assessed by analyzing correlation of signals between duplicate spots printed for each protein.

### Statistical analysis

Sample sizes were selected for in vivo studies based on prior experience with this model and the effects of pharmacological intervention to alter the critical stroke-related phenotypes collected in the study. Sample sizes for in vitro studies reflected the reduced inter-individual variation of cellular phenotypes arising from a single genetic change, compared with in vivo phenotypes. No animals or cellular samples were excluded from the study. Care was taken to ensure selection of animals and samples collected from animals to avoid litter effects. The genetic nature of the “treatment” to be tested did not allow randomization: animals were assigned to study groups based on congenic status. Blinding was performed for histological analysis. The remainder of the study could not be performed blinded. Comparison of data from SHR-A3 and congenic rats was analyzed using the Student’s *t*-test using the Prism 7 software (GraphPad Software, La Jolla, CA). ANOVA with Tukey’s post-hoc test was used to compare data from multiple groups. Two-way tests of significance were applied and a value of *p* < 0.05 was considered statistically significant, with *n* = 7–10 independent animals per group and 5–7 replicates for in vitro studies per group. In general, SHR-A3 group variances in the in vivo experiments were greater than SHR-A3(Stim1 B2). This is to be expected if the genetic difference between the strains creates two distinct populations with respect to the emergence of disease. The group variances in in vitro phenotypes were similar.

## Results

### Introgressed congenic segment in SHR-A3(*Stim1* B2)

**Fig. 1:**
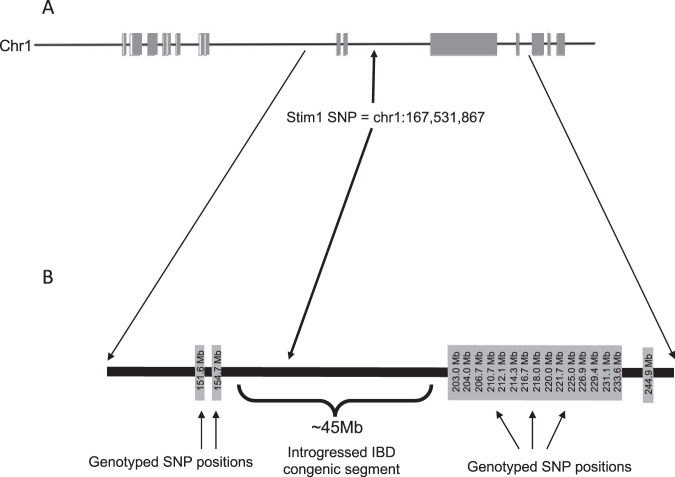
Map of introgressed congenic segment. Across the entire genome, SHR-A3 and SHR-B2 are 87% genetically identical by descent (IBD). Regions of non-IBD show a haploblock structure. **a** Blocks of non-IBD on chromosome 1 surround a ~45 Mb block of IBD in which the genome sequence of SHR-A3 and SHR-B2 are identical. The only SNP in this region affecting a coding sequence occurs in SHR-A3 and affects *Stim1*, producing a premature stop codon. **b** The IBD block containing the *Stim1* SNP was transferred from SHR-B2 to SHR-A3 by genome wide marker-assisted backcrossing. The positions of SNP markers in each of the blocks of non-identity surrounding *Stim1* are indicated.

Figure 1 shows the chromosome-scale map of divergence between SHR-A3 and SHR-B2 on rat chromosome 1. The block structures represent regions of the chromosome that are not identical and are derived from two different recent ancestors. The positions of single nucleotide polymorphisms used to demonstrate that the congenic line SHR-A3(*Stim1*B2) had retained all SHR-A3 makers in these blocks. A targeted genotyping assay was used to verify that *Stim1* had been converted from the mutant SHR-A3 to the wild type SHR-B2 *Stim1* allele in the congenic line.

### Effect of *Stim1* rescue on immune cell function

**Fig. 2:**
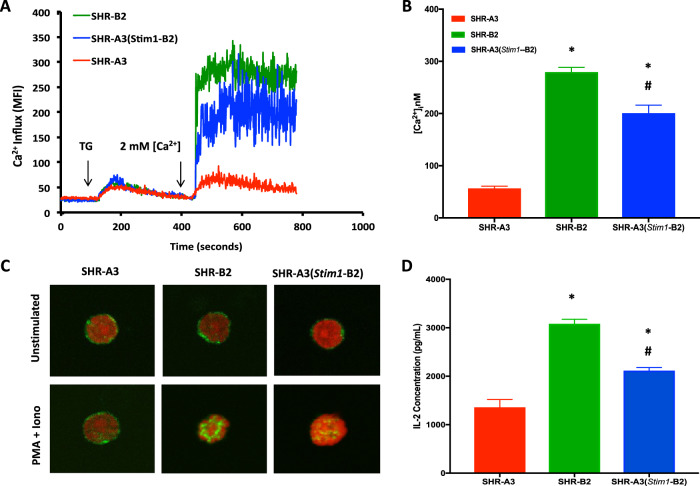
Effect of the *Stim1* mutation on SOCE and NFAT activation. **a** Average time course for [Ca^2+^]_i_ influx in response to store-depletion by thapsigargin (TG, 2 μmol/L) followed by Ca^2+^ re-addition to induce SOCE in peripheral blood mononuclear cells (PBMC). **b** Summary graph of sustained phases of TG-induced Ca^2+^ influx. **c** Confocal microscopy of NFATc1 nuclear translocation in SHR-A3, SHR-B2 and SHR-A3(*Stim1*-B2) T cells stimulated for 60 min with PMA (10 nmol/L) and Ionomycin (2 μmol/L). Green indicates NFATc1 staining and red indicates nuclear stain using Draq5. **d** Quantification of IL-2 production by CD3^+^ T cells from SHR-A3, SHR-B2, and SHR-A3(*Stim1*-B2) rats in response to PMA (10 nmol/L) and Ionomycin (2 μmol/L) for 4 h. Asterisk (*) indicates *P* < 0.05 vs SHR-A3 and hash (#) indicates *P* < 0.05 vs SHR-B2 with *n* = 5 per group.

We have previously shown that SHR-A3 *Stim1* premature stop codon does not result in nonsense-mediated RNA decay, but rather in expression of a truncated STIM1 protein lacking key function domains in the C terminus that are involved in coordination of SOCE [13]. We examined whether this effects SOCE in peripheral blood mononuclear cells (PBMC) by assessing SOCE using fluorescent intracellular calcium ion concentration measurements. Endoplasmic reticulum (ER) calcium stores were depleted by thapsigargin (TG) treatment. *Stim1* is an ER [Ca^2+^] detector and this treatment primes *Stim1* to activate entry of extracellular calcium in response to *Stim1*-mediated gating of the plasma membrane SOCE calcium channel encoded by Orai1. The presence of a natural truncating mutation in *Stim1* in SHR-A3 is associated with a substantial deficit in SOCE, compared with SHR-B2 (Fig. 2a, b). This deficit is significantly reduced in PBMC by rescue of the *Stim1* mutation by congenic substitution with the wild type *Stim1* allele from SHR-B2 (Fig. 2a, b). A subset of cells pre-treated with the Orai1 channel blocker Pyr6 (5 μmol/L) for 15 min confirmed the dependence of observed SOCE on STIM1-Orai1 interaction (data not shown).

The calcium signal generated by SOCE in lymphocytes is essential for T lymphocyte activation. This results from activation of the calcium-dependent protein phosphatase, calcineurin, and the subsequent dephosphorylation and nuclear localization of the transcriptional regulator, nuclear factor of activated T lymphocytes, (NFAT). Nuclear localization of NFAT in response to calcium activation of T lymphocytes from SHR-A3 was severely reduced compared with SHR-B2, but was restored in SHR-A3(*Stim1*-B2) congenic animals (Fig. 2c). Similarly, we observed difference in secretion of the key T lymphocyte cytokine that is upregulated in response to NFAT, interleukin 2 (IL-2). Calcium activation resulted in significantly smaller increments in IL-2 production in SHR-A3 T lymphocytes than in lymphocytes from either SHR-B2 or SHR-A3(*Stim1*-B2) (Fig. 2d).

### Effect of *Stim1* gene rescue on stroke

**Fig. 3:**
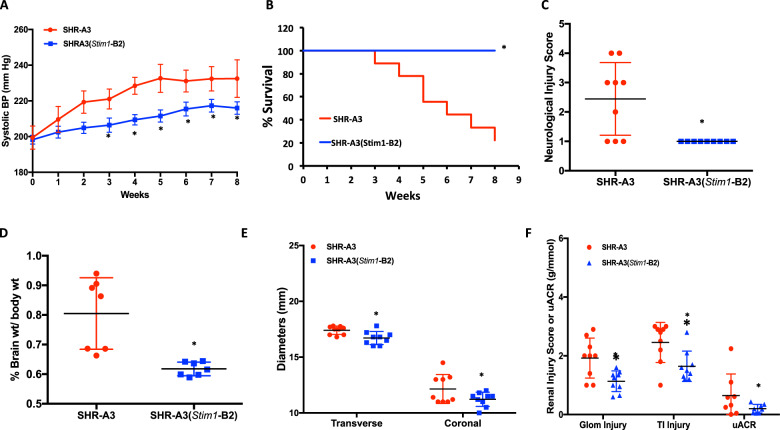
Effect of variation in *Stim1* on stroke susceptibility. SHR-A3 and SHR-A3(*Stim1*-B2) rats were maintained for 8 weeks on a stroke-inducing diet (1% NaCl in drinking water) starting at 20 weeks of age. **a** Weekly systolic blood pressure measured by telemetry for the 8 weeks of salt loading. **b** Kaplan Meier curve for survival over 8 weeks of salt loading. **c** The severity of neurological symptoms scored using the Yamori classification scheme at euthanasia in stroke sign positive rats, or at the end of the treatment protocol in surviving rats. Brain morphometrics: **d** extent of cerebral edema as reflected in brain weights normalized to initial body weights (prior to salt loading), **e** extent of cerebral edema as reflected in transverse and coronal brain diameters. Asterisk (*) indicates *P* < 0.05 vs SHR-A3 with *n* = 9 (SHR-A3), *n* = 10 (SHR-A3(*Stim1*-B2)). **f** Measures of renal injury in salt loaded SHR-A3 and SHR-A3(Stim1 B2). Glomerular (Glom) and tubulointerstitial (TI) histological injury and urinary albumin/creatine ratio (uACR) were assessed using our published methods [11, 49]. Hash (#) indicates *P* < 0.05 vs SHR-A3 with *n* = 9 (both SHR-A3 and (SHR-A3(*Stim1*-B2)).

**Fig. 4:**
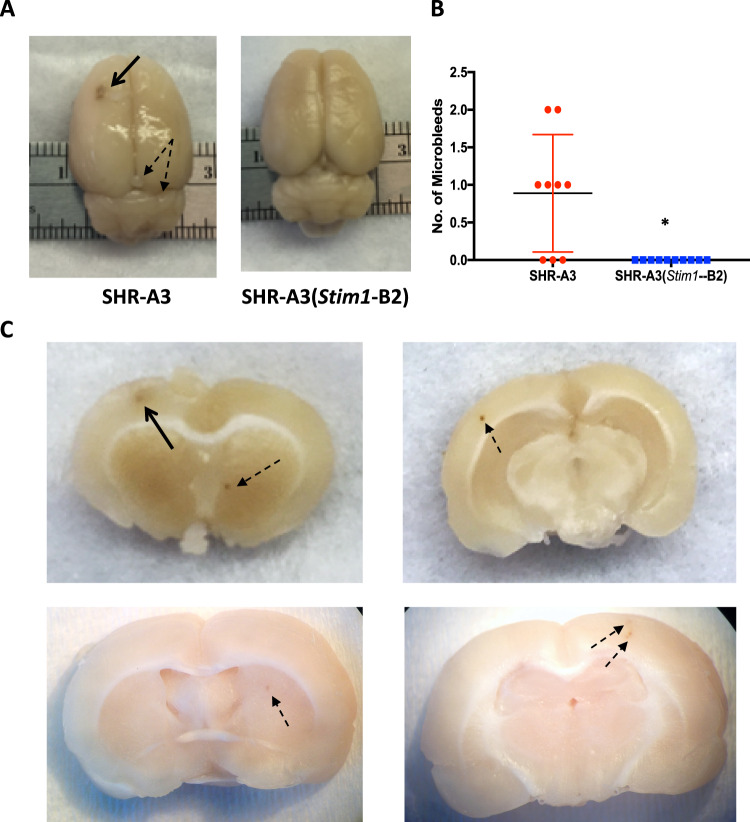
Stroke phenotype in salt-loaded rats. SHR-A3 and SHR-A3(*Stim1*-B2) rats were maintained for 8 weeks on a stroke-inducing diet (1% NaCl in drinking water) starting at 20 weeks of age. **a** Gross morphology of the brains from SHR-A3 rats revealed global cerebral edema, evidenced by the filling in of major brain sulci (*dashed arrows*) in SHR-A3 rats. A hemorrhage is also visible (*solid arrow*). **b** Quantification of microbleeds in SHR-A3 and SHR-A3(*Stim1*-B2) rats. **c** Representative image of a brain slices showing a hemorrhagic lesions (*solid arrow*) and microbleeds (*dashed arrows*) in SHR-A3 rats. Hash (#) indicates *P* < 0.05 vs SHR-A3 with *n* = 9 (SHR-A3), *n* = 10 (SHR-A3(*Stim1*-B2)).

To assess whether the *Stim1* mutation contributes to genetic susceptibility to cerebrovascular disease, SHR-A3 and SHR-A3(*Stim1*-B2) rats were placed on a stroke-inducing increase in salt intake (1% NaCl in drinking water) for 8 weeks starting at 20 weeks of age [18]. Blood pressure elevation accompanies salt loading in SHR-A3 and may influence emergence of cerebrovascular disease. We observed a correlation between systolic blood pressure reaching around 230 mmHg and the emergence of cerebrovascular disease in salt-loaded SHR-A3 animals that was followed rapidly by death or euthanasia. SHR-A3 group mean blood pressures plateaued at 5 weeks of salt loading as the most severely hypertensive animals began to be removed by death or euthanasia from the cohort. The progressive BP increase in salt-loaded SHR-A3 rats was blunted in SHR-A3(*Stim1*-B2) rats (Fig. 3a). Compared with SHR-A3 rats, *Stim1*-rescued SHR-A3 had markedly improved survival. By the end of the treatment period only two of nine SHR-A3 were alive, seven animals either died with evident cerebrovascular disease or were euthanized. In contrast, ten of ten SHR-A3(*Stim1*-B2) remained at this time point (Fig. 3b). Neurological deficit (Yamori) scores [8, 18] were assessed weekly and at termination (week of death, euthanasia or end of study period) were normal in SHR-A3(*Stim1*-B2) animals, but elevated in SHR-A3 animals (Fig. 3c). Salt loading resulted in significant cerebral edema in SHR-A3 rats, but not in the SHR-A3(*Stim1*-B2) rats (Fig. 3d, e). Microbleeds, which predict future cerebral hemorrhages in humans [19–21], were observed in sections of brains in 5 of 9 SHR-A3 rats and major hemorrhages were found in 3 of 9 animals (Fig. 4a–c) primarily in the cortex, subcortical white matter, and striatum. These lesions were completely absent in the salt-loaded SHR-A3(*Stim1*-B2) group. Our findings indicate that, in SHR-A3, a single base change in *Stim1* creates stroke susceptibility in the presence of hypertension.

### Effect of *Stim1* gene rescue on renal injury during salt loading

The development of stroke in SHR-A3 occurs after the emergence of progressive hypertensive renal injury. Efforts to separate the pathogenic mechanism of renal injury from stroke have not been fully successful [22] and this may reflect a shared mechanism in which genetic susceptibility to end-organ injury in both kidney and brain arises from genetic variation that acts on both. Assessment of histological measures of renal injury in salt-loaded SHR-A3(Stim1 B2) congenic line in comparion with SHR-A3 under the same conditions indicates that Stim1 participates in the pathogenesis of renal injury in addition to its contribution to stroke susceptibility (Fig. 3f). The contribution of defective Stim1 function to renal injury may accelerate the elevation of blood pressure during salt loading in SHR-A3 as a result of amplified renal injury and the effect of elevated blood pressure may be a primary mechanisms of stroke pathogenesis. At present, our studies are unable to further resolve this possibility.

### Emergence of autoreactive antibodies in salt-loaded SHR-A3 and SHR-A3(*Stim1*-B2)

Deficiency of *Stim1* is associated with autoantibody formation [23]. Autoantibodies can contribute to the emergence of disease in a number of ways. This can include binding to host proteins and limiting the function of these antigenic proteins. In order to uncover antigens that are potential targets for pathogenic antibodies in SHR-A3 serum we examined fluorescence signals attributable to IgM and IgG binding to the HuProt v3.1 protein array, a printed array containing nearly 20,000 unique full length, recombinant human proteins expressed in a eukaryotic host [24]. Serum was pooled from six animals each, representing the following groups: 16-week-old SHR-A3 without salt loading; 28-week-old salt-loaded SHR-A3(*Stim1*-B2); and salt-loaded SHR-A3 animals at collected at 28 weeks of age (*n* = 2) or at euthanasia due to salt loading-induced cerebrovascular disease (*n* = 4).

We first sorted the signals (normalized as Z scores) obtained with pooled serum from salt-loaded SHR-A3 animals to identify recombinant human proteins to which the largest Z scores were obtained, eliminating all antigens that generated Z scores of less than 3 (per array manufacturer’s recommendation) and selecting the 35 antigens generating the largest signals. This was done for both IgM and IgG signals independently. We then compared the Z scores obtained for these antigens using pooled serum from 16-week-old SHR-A3 consuming normal salt intake. We eliminated antigens that had higher Z scores in 16-week-old animals than in the salt-loaded animals (14 IgM and 4 IgG signals). We then examined binding of IgM and IgG to the array in similar serum pools from SHR-A3(*Stim1*-B2) that were obtained after salt loading. We identified those antigens that had Z scores that were reduced in salt-loaded congenic animals compared to the levels observed in salt-loaded SHR-A3.

There was functional overlap among the antigens producing both IgG and IgM signals that were lower in SHR-A3(*Stim1*-B2) congenic animals than in SHR-A3 (Table 1). Among the seven antigens identified with reactive IgM and IgG, two antigens (Hspa1L and Hspa6) were members of the HSP70 family of stress-responsive heat shock proteins. Human Hspa1L is closely homologous to rat Hspa1L (Blast score 1254). Hspa6 is a human protein that is not found in the rat or mouse genome but has high homology to rat Hspa1a (Blast homology score = 1090). Other antigens with both IgM and IgG reactivity that are elevated in salt-loaded SHR-A3, but reduced in the SHR-A3(*Stim1*-B2) congenic line include Letm1, Lrrfip1, and Eaf1, a group of proteins involved in Wnt/beta-catenin signaling that consequently may be related to brain vascularization and blood-brain barrier function [25–28].

**Table 1:** Antigens producing IgM and IgG signals on the HuProt array.

Name	ID	IgM Z score			IgG Z score		
		SHR-A3 16 weeks (no salt load)	SHR-A3 28 weeks	SHR-A3 (Stim1 SHR-B2) 28 weeks	SHR-A3 16 weeks (no salt load)	SHR-A3 28 weeks	SHR-A3 (Stim1 SHR-B2) 28 weeks
HSPA1L	JHU04079.P043D09	22.31	50.18	14.11	8.55	23.82	6.44
LETM1	JHU01877.P176F10	8.01	29.57	9.00	2.30	10.45	3.32
EAF1	JHU05677.P060G08	10.82	19.47	18.58	6.28	10.32	9.89
GARS	JHU11709.P122C09	3.53	19.46	6.96	7.44	8.42	2.84
FAM131C	JHU05680.P060H09	10.78	13.60	10.82	4.80	8.09	6.95
LRRFIP1	JHU25415.P241B12	−0.27	11.96	−0.24	−0.14	12.36	−0.17
HSPA6	JHU18279.P210A07	1.42	10.86	2.15	0.74	7.43	1.30

## Discussion

The risk of cerebrovascular disease is not uniformly distributed in the hypertensive population, suggesting that elevated blood pressure is necessary, but may not be sufficient to cause disease. Heritability estimates indicate genetic variation can contribute to disease risk [29–31], and SHR lines contrasting in stroke susceptibility illustrate how inheritance of genetic variation can create stroke susceptibility [6, 10]. Identification of genetic variation contributing to stroke heritability offers the opportunity for mechanistic insights into stroke pathogenesis. Large-scale human population genetics studies intended to uncover the genetic variants that create cerebrovascular injury have uncovered numerous gene associations, but these are of limited effect size and offer little pathogenic insight [4, 32]. Genetically hypertensive inbred rat strains provide the advantages of reduced genetic and pathogenic complexity, as hypertensive SHR lines share an overlapping set of hypertension alleles [17], and environmental variation that is limited by shared housing and breeding conditions.

Previous studies of SHR-A3 have indicated a stroke susceptibility locus in the region of Chr1 containing the Stim1 mutation. Rubattu et al. performed an F2 intercross study and identified linkage to stroke centered on a microsatellite marker located ~11 Mb from the Stim1 mutation [12] and created congenic lines to support the presence of a stroke susceptibility locus in SHR-A3 encompassing ~50 Mb and including the location of Stim1 [33]. Gandolgor et al. also mapped stroke susceptibility in a cross between SHR-A3 and a stroke-resistant SHR line and localized a susceptibility region on Chromosome 1 that does not overlap with the Stim1 locus [34].

Rare homozygous deletion mutations in *Stim1* in humans create an immune phenotype including both T lymphocyte immunodeficiency and antibody-mediated autoimmune disease [23, 35]. Spontaneous autoantibody-mediated tissue injury occurs when *Stim1* and *Stim2* are deleted in mouse CD4^+^ T cells [16]. We have reported that SHR-A3 has a naturally occurring truncating mutation in the *Stim1* gene [13] that reduces SOCE and show here that this has important effects to reduce T helper cell activation. Creation of the congenic SHR-A3(*Stim1*-B2) line demonstrates that rescue of defective *Stim1* function prevents development of cerebrovascular disease in SHR-A3 while restoring T helper cell functions. *Stim* deficiency limits the ability of T helper cells to provide effective help to B cells as the latter undergo antibody affinity maturation [16, 36]. This results in premature termination of affinity maturation and the development of antibody-secreting cells that generate self-reactive antibodies. This suggests a possible participation of antibody-dependent disease mechanisms in stroke in SHR-A3.

While comparable mutations in *Stim1* to that we have described here can be expected to be very rare in humans, the functional role of *Stim1* in lymphocyte calcium signaling places it in a pathway that alters T- and B-cell interactions in which human genetic variations are well recognized. These variations predispose to autoimmune disease and suggests the possibility that such variation may contribute to stroke risk in the presence of hypertension in human populations. The range of genes participating in such immune signaling variation has been recently surveyed and warrant further investigation in stroke occurring in genetically predisposed humans experiencing elevated blood pressure [37].

We examined whether genetic variation in *Stim1* contributes a difference in blood pressure. By implanting animals prior to salt loading we determined that SHR-A3(*Stim1*-B2) congenic animals had similar levels of blood pressure to SHR-A3. Emergence of end-organ injury after salt loading in SHR-A3 includes renal injury, which may account for part or all of the additional elevation in blood pressure in SHR-A3 [18]. The elevation in blood pressure associated with salt loading in SHR-A3(*Stim1-*B2) congenics was lower than SHR-A3. It is possible that one determinant of stroke and cerebrovascular disease occurrence in SHR-A3 is the level of blood pressure attained and that beneficial effects of congenic substitution include a benefit of reduced renal injury and reduced effect of salt loading to further amplify hypertension. To this extent it is not possible to determine whether antibody mechanisms have their primary effect on renal injury or cerebrovascular injury or whether both contribute to disease.

The application of HuProt array technology to the identification of autoreactive antibodies in rats provides a discovery approach to investigate the possible emergence and reactivity of pathogenic antibodies relevant to cerebrovascular disease. The results obtained appear to be of potential biological relevance in revealing the presence of potentially self-reactive antibodies that target antigens that recognize proteins with functions linked to cerebrovascular disease. It is well known that cerebral ischemia induces expression of HSP70 family stress proteins and that this has protective effects against ischemic injury [38–41]. This is an important adaptation to ischemic stress that may arise as blood pressure begins to exceed the range of autoregulatory control of blood flow in the brain [42]. Adaptation to ischemic stress is potentially compromised by the formation of neutralizing autoantibodies to HSP70 protein. Similarly, three antigens for which autoantibodies were recognized participate in Wnt/β-catenin signaling. Lrrfip1 is a brain protein upregulated in cerebral ischemia that activates β-catenin and regulates pro-survival pathways [25, 43]. Eaf1 binds to β-catenin and regulates Wnt signaling [26]. Letm1 downregulation leads to downregulation of β-catenin [27, 44]. All three genes are expressed in brain. A very large IgG signal was detected for Fabp4, but was not present in the IgM signal (Supplementary data). The Fabp4 IgG signal was much reduced in the SHR-A3(*Stim1*-B2) congenic line. Fabp4 is a marker of stroke risk [45], a prognostic indicator of stroke outcome [46, 47] and is a pro-angiogenic factor expressed in vascular endothelial cells [48]. The HuProt array poses limitations: first, the arrayed proteins are not rat proteins. This may enrich the discovered autoantigens for proteins that are most highly conserved between rats and humans. Second, the arrays are estimated to contain ~80% of the human proteome, so antigens not represented will not be detected by SHR autoantibodies. Finally, pathogenic antibodies whose targets are not proteins cannot be uncovered.

In conclusion, we have identified genetic variation in SHR-A3 affecting *Stim1*. We have shown that this variation contributes substantially to risk of cerebrovascular disease *Stim1* defects may participate in the formation of autoantibodies that contribute directly or indirectly to the emergence of cerebrovascular disease. Congenic substitution and *Stim1* are associated with reduction in the level of serum IgM and IgG reactive to targets that may be involved in adaptation to cerebral ischemia occurring as blood pressure exceeds the cerebral autoregulatory range.

## Supplementary information

HuProt supplemental data
